# Converting Galactose into the Rare Sugar Talose with Cellobiose 2-Epimerase as Biocatalyst

**DOI:** 10.3390/molecules23102519

**Published:** 2018-10-01

**Authors:** Stevie Van Overtveldt, Ophelia Gevaert, Martijn Cherlet, Koen Beerens, Tom Desmet

**Affiliations:** Centre for Synthetic Biology, Faculty of Bioscience Engineering, Ghent University, Coupure Links 653, 9000 Gent, Belgium; stevie.vanovertveldt@ugent.be (S.V.O.); ophelia.gevaert@ugent.be (O.G.); martijn.cherlet@telenet.be (M.C.); koen.beerens@ugent.be (K.B.)

**Keywords:** biocatalysis, cellobiose 2-epimerase, rare sugars, talose, enzyme engineering

## Abstract

Cellobiose 2-epimerase from *Rhodothermus marinus* (*Rm*CE) reversibly converts a glucose residue to a mannose residue at the reducing end of β-1,4-linked oligosaccharides. In this study, the monosaccharide specificity of *Rm*CE has been mapped and the synthesis of d-talose from d-galactose was discovered, a reaction not yet known to occur in nature. Moreover, the conversion is industrially relevant, as talose and its derivatives have been reported to possess important antimicrobial and anti-inflammatory properties. As the enzyme also catalyzes the keto-aldo isomerization of galactose to tagatose as a minor side reaction, the purity of talose was found to decrease over time. After process optimization, 23 g/L of talose could be obtained with a product purity of 86% and a yield of 8.5% (starting from 4 g (24 mmol) of galactose). However, higher purities and concentrations can be reached by decreasing and increasing the reaction time, respectively. In addition, two engineering attempts have also been performed. First, a mutant library of *Rm*CE was created to try and increase the activity on monosaccharide substrates. Next, two residues from *Rm*CE were introduced in the cellobiose 2-epimerase from *Caldicellulosiruptor saccharolyticus* (*Cs*CE) (S99M/Q371F), increasing the *k*_cat_ twofold.

## 1. Introduction

Carbohydrate epimerases are a subclass of isomerases capable of inverting the configuration of hydroxyl groups of both substituted (phosphate and nucleotide diphosphate) and unsubstituted sugars [1]. During recent years, more attention has been drawn towards the latter, evidenced by the growing number of characterized enzymes and industrial applications [2,3,4,5,6,7]. d-tagatose 3-epimerase, for example, plays an important role in the production of d-psicose (showing potential as no-calorie sweetener and antidiabetic [8]) from sucrose where it is combined with two other enzymes in a one-pot cascade reaction [9]. Another example is cellobiose 2-epimerase, which catalyzes the interconversion of glucose to mannose at the reducing end of disaccharides (e.g., cellobiose and lactose) and oligosaccharides (e.g., cellotriose and cellotetraose) [10]. Common applications are the production of epilactose starting from milk ultrafiltrate containing lactose or the production of lactulose from whey powder. Epilactose is regarded as a new prebiotic and lactulose is a potential low-calorie sweetener that could substitute for lactose in the food industry [11,12]. 

Even though cellobiose 2-epimerase enzymes have a rather relaxed substrate specificity, their activity is typically limited to oligosaccharides [5]. Until today, only the enzyme from *Caldicellulosiruptor saccharolyticus *(*Cs*CE) has been shown to also accept monosaccharides as substrate, a feature that was exploited for the production of mannose from glucose [4]. However, similar enzymes might still exist as not all current representatives have been screened for their monosaccharide activity and, therefore, it is possible that interesting epimerase reactions might have gone unnoticed. This paper focuses on the cellobiose 2-epimerase from *Rhodothermus marinus*, which has already been characterized regarding activity on oligosaccharides and has displayed the ability to produce epilactose from lactose efficiently. Furthermore, the enzyme was shown to be stable at temperatures up to 80 °C, making it highly attractive for potential production processes [5]. In this research, the substrate specificity of *Rm*CE was explored with a particular emphasis on the C2 epimerization of galactose to talose, a rare sugar with potential applications in the pharmaceutical industry [13,14,15,16]. It can, for example, serve as a precursor molecule for several antibiotics of which the best example is Caminoside A, an antimicrobial compound isolated from the sponge *Caminus sphaeroconia*, containing a 6-deoxytalose group [13]. Moreover, the O2 and O3-methylated forms have been shown to be submillimolar inhibitors of galactose-binding galectin-4 and galectin-8, proteins which are involved in inflammation and cancer [17].

Currently, talose can only be produced by a rather inefficient chemical process, requiring either high amounts of dibutyltin oxide [18] or five consecutive chemical steps and a lot of solvents [19]. Therefore, an efficient biochemical route would provide a clear added value to the production of talose.

## 2. Results

### 2.1. Substrate Specificity Analysis of Rhodothermus marinus (RmCE) Revealed One-Step Conversion from d-Galactose to d-Talose

The specificity of the target cellobiose 2-epimerase was evaluated on a range of substrates, comprising d-hexoses, d-pentoses and an l-pentose (Table 1). The analysis revealed that *Rm*CE is able to accept various monosaccharides, with a specific activity that is about 1% of that on cellobiose. Interestingly, the activity on galactose was found to be similar to that of mannose, which can be regarded as the naturally preferred monosaccharide. However, further kinetic analysis showed that the affinity for galactose is considerably lower (*K*_m_ of 1.04 and 0.45 M, respectively) whereas the *k*_cat_ is roughly the same (5 s^−1^) on both of these substrates (Table 2). While the mannose to glucose reaction was already described for the *Cs*CE enzyme, it is the first time that a one-enzyme conversion from galactose to the rare sugar talose is demonstrated.

### 2.2. Production of d-Talose

**Figure 1:**
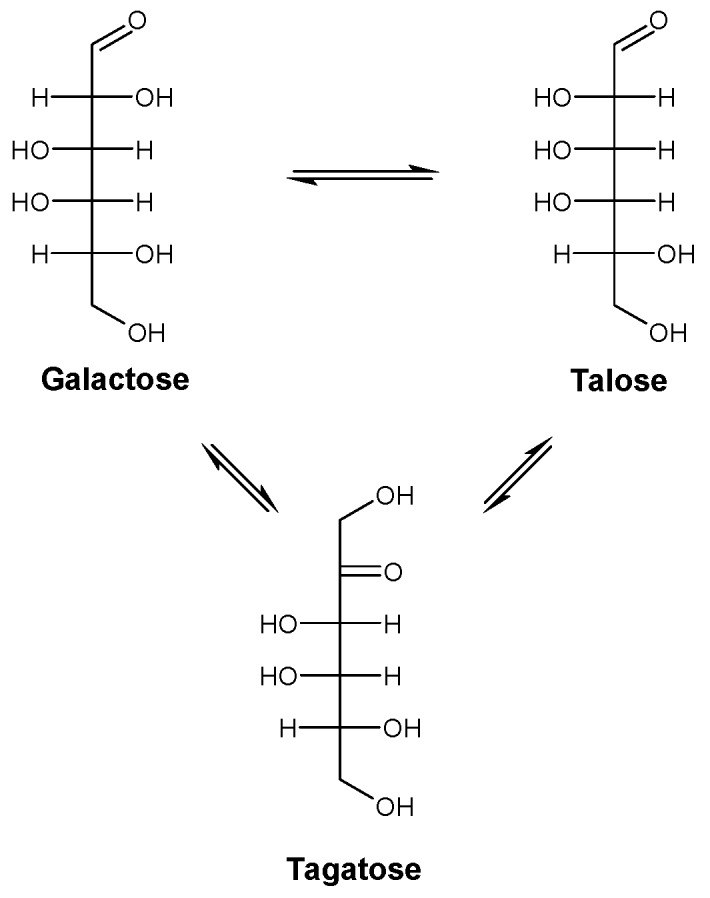
Galactose to talose conversion by *Rm*CE also generates the isomerization product tagatose.

**Figure 2:**
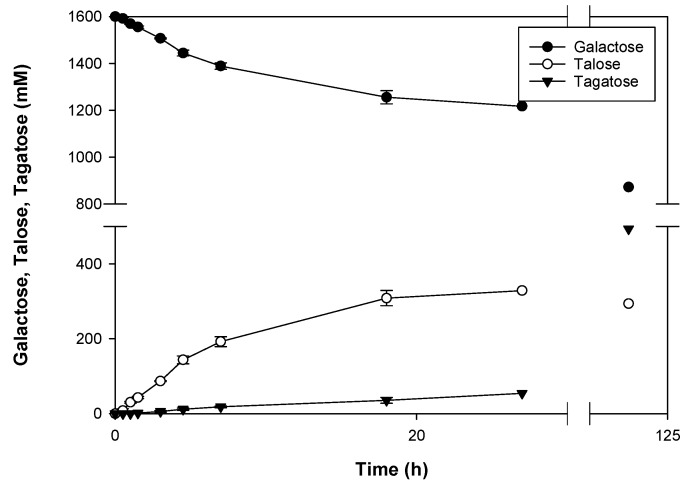
Time-course representation of galactose to talose/tagatose conversion. The reaction was performed with 1.6 M of galactose at pH 6.3 and monitored for 27 h at 70 °C. During the first hours, talose was being formed time-dependently, reaching a maximum conversion of about 20% after overnight incubation. Formation of tagatose seemed to lag in the beginning of the reaction but increases linearly after one hour. The tagatose equilibrium was only reached after 120 h, during which extra enzyme was added to speed up the reaction. Data represent the means of three experiments with standard deviation (until 18 h).

During prolonged incubations with galactose, the keto-aldo isomer tagatose (Figure 1) also started to appear (Figure 2), as was observed during the production of mannose from glucose by *Cs*CE [4]. As a consequence, the purity of the produced talose slowly decreased during the reaction, with the final ratio of galactose, tagatose and talose being 53:28:19 at equilibrium, demonstrating that galactose is the most stable isomer. Interestingly, the talose equilibrium was reached first, while the concentration of tagatose kept increasing until about 28% yield was reached. The presence of the isomerization product tagatose was also spotted during the kinetic analysis (see previous paragraph) but was neglibly low and, therefore, not incorporated in the calculations.

In order to find the optimal conditions for the production of talose, various substrate concentrations were evaluated and the product purity (expressed as the amount of talose to the total amount of talose and tagatose) was monitored over time (Table S1). High substrate concentrations were found to yield the highest purity during longer incubation times, as it takes longer to reach the talose equilibrium. Moreover, these substrate concentrations are the most attractive as they also give rise to the highest product concentrations. 

When focusing on the reaction profile of 1.6 M galactose, it becomes clear that purity is >99% during the first hour and then drops during the next hours. However, the total amount of talose keeps increasing during the course of the reaction. As a consequence, the optimal reaction time is dependent on the specific wishes of talose production. If a very pure product is desired for pharmaceutical applications, a shorter incubation time is preferred. In contrast, if purity is less important, the reaction time could be extended to maximize product yield (Table 3). 

Next, the production of talose was performed at a larger scale, starting from 288 g/L of galactose. This generated 24.3 g/L of talose after 4.5 h, which corresponds to a molar yield of about 8.5%. The relatively low degree of conversion is mainly caused by the superior stability of galactose, as reflected by these compounds’ equilibrium ratio (see above). However, the remaining galactose could simply be removed by a yeast treatment with *Kluyveromyces marxianus* (Figure S1) [20,21]. This selective digestion of a 2-epimer by micro-organisms has already been demonstrated in the production of *N*-glycolylneuraminic acid [22,23]. After 96 hours incubation, less than 1% of galactose was still present, and 23 g/L (1.92 mmol) of talose could be recovered at a purity of 86% (containing 12% of tagatose and trace amounts of galactose and the yeast metabolite glycerol) (Figure S2).

### 2.3. Engineering of RmCE to Increase Activity on Monosaccharide Substrates

Given the clear application potential of the *R. marinus* cellobiose 2-epimerase, the opportunity to increase the affinity towards monosaccharides by enzyme engineering was explored. Mannose to glucose conversion was used as case study, because the detection of glucose can be performed in more high-throughput manner by making use of the glucose oxidase-peroxidase (GOD-POD) assay [24]. 

**Figure 3:**
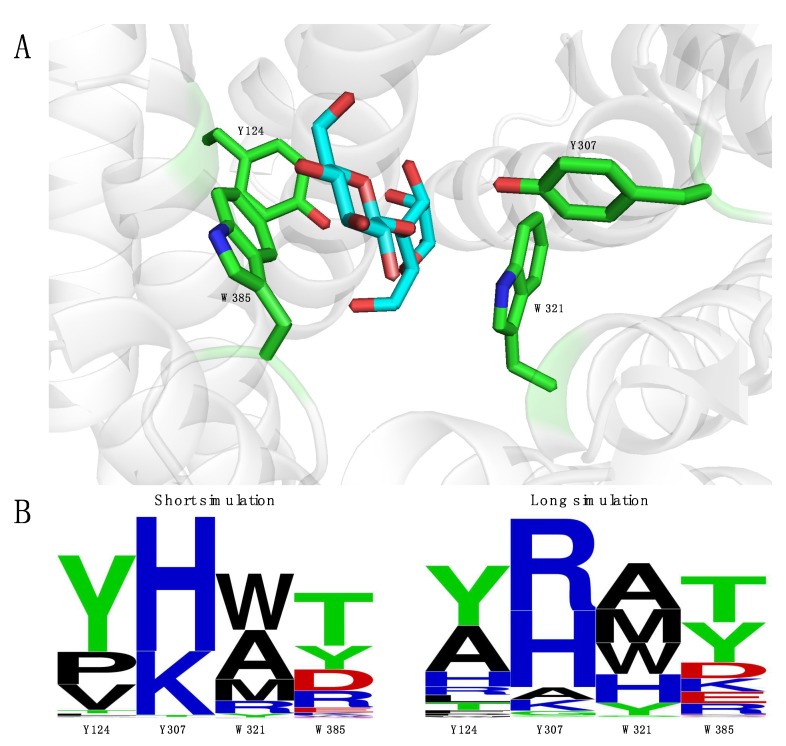
(**A**) Active site representation of *Rm*CE (PDB code: 3WKG) displaying the four residues (Tyr-124, Tyr-307, Trp-321, Trp-385) that were targeted for mutagenesis [10]. The substrate glucosylmannose is colored by element cyan (carbon in cyan, oxygen in red) while the residues were colored by element green (carbon in green, oxygen in red, nitrogen in blue). (**B**) Sequence motifs for both the short simulation and long simulation mutant library. Motifs were created by the online software Weblogo [29].

The active site was explored and four positions appeared interesting for mutagenesis: Tyr-307 and Trp-321 had no important function in catalysis but were nonetheless oriented towards the substrate; Tyr-124 already makes a hydrogen bond with the O2 of mannose but might still be improved; Trp-385 displays a stacking interaction with the non-reducing end of cellobiose, but since this part is not present when mannose is supplied as substrate, this provides an opportunity to create a new interaction (Figure 3A). Simultaneously saturating all four positions would leave us with the impossible task of screening roughly half a million mutants (3 × 20^4^) [25]. Therefore, the Rosetta software suite was considered, as it is able to seriously diminish library size by suggesting mutations that increase affinity between enzyme and substrate [26,27,28]. 

Two separate Rosetta analyses were performed, the only difference being the starting structures. They both originated from snapshots of molecular dynamics simulations with a duration of 0.1 ns and 5 ns, respectively. The corresponding mutant libraries were then designed based on amino acids occurring in more than 5% of the sequences on a given position (Figure S3). Strikingly, some differences occur between the motives (Figure 3B), evidencing that different simulation times can provide alternative amino acids on positions of interest. However, neither of these libraries delivered improved mutants. The percentage of inactive mutants was comparable between both libraries.

### 2.4. Introducing Two RmCE Residues in Caldicellulosiruptor saccharolyticus (CsCE) Led to A Twofold k_cat_ Increase

As mentioned earlier, the only other cellobiose 2-epimerase showing significant activity towards monosaccharides is *Cs*CE. When comparing the substrate specificities of these enzymes, it becomes clear that some differences occur: *Cs*CE displays a higher activity towards d-xylose and d-lyxose and *Rm*CE is active on galactose, an activity not yet reported for *Cs*CE. Moreover, the *k*_cat_ of *Rm*CE for mannose is significantly higher than that of *Cs*CE (5.4 and 0.7 s^−1^, respectively) [4,5].

**Figure 4:**
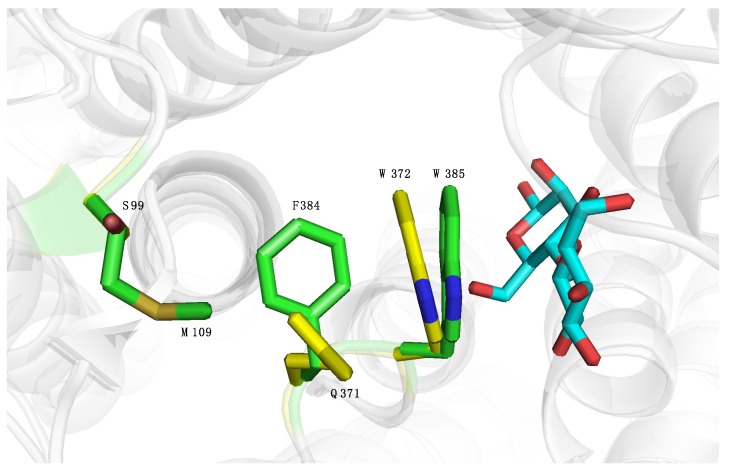
The catalytic Trp residue in the active sites of *Rm*CE (carbon in green, nitrogen in blue, sulfur in yellow) (PDB code: 3WKG) and *Cs*CE (carbon in yellow, nitrogen in blue, oxygen in red) (PDB code: 4Z4J) has a slightly different orientation towards the glucose moiety of glucosylmannose (carbon in cyan, oxygen in red). In the active site of *Rm*CE, Trp-385 is oriented closer to glucose, probably due to the accompanying resides Phe-384 and Met-109.

Analyzing the crystal structures of *Rm*CE and *Cs*CE revealed that one of the common first shell residues is oriented differently: in *Rm*CE, Trp-385 (in *Cs*CE Trp-372) is closer to the non-reducing end of cellobiose. This could be explained by the presence of the bulky amino acid Phe-384, altering the angle of Trp-385 towards the glucose moiety. This in turn seems correlated to the neighbouring amino acid Met-109, orienting Phe-384 (Figure 4). To validate whether the orientation of Trp-385 had any influence on the activity, a *Cs*CE double mutant S99M/Q371F was created that displayed two times higher specific activity towards mannose than the wild-type *Cs*CE. Kinetic analysis showed that this could be attributed to an increase in *k*_cat_ of the mutant (7.5 versus 3.8 s^−1^), while the *K*_m_ was not significantly affected (Table 4). This can be explained by the higher *k*_cat_ value of wild-type *Rm*CE, which apparently can be transferred towards *Cs*CE by mutating these two residues. However, it is not clear whether other residues can still increase the *k*_cat_ of *Cs*CE: according to our measurements the *k*_cat_ of *Cs*CE is only slightly lower than *Rm*CE, but literature reports a significantly lower *k*_cat_ (0.7 s^−1^) and *K*_m_ (52 mM) for *Cs*CE [4,5]. 

## 3. Discussion

Most cellobiose 2-epimerase enzymes are characterized by a broad substrate specificity meaning that they can be used for the production of several important molecules (epilactose, mannose, lactulose) from their cheaper counterparts [4,11,12,30,31,32]. The specificity of cellobiose 2-epimerase from *Rhodothermus marinus* was explored further and revealed side-activity on several monosaccharides comprising the conversion of d-glucose to d-mannose (comparable to *Cs*CE) and d-galactose to d-talose (a new reaction). As a consequence, the previous assumption that CE enzymes are only active on β-1,4-substituted sugars might be questioned and more cellobiose 2-epimerases might still exist in nature that exhibit monosaccharide activity. In order to further clarify the activity of CE enzymes on monosaccharides, galactose was docked in the active site of *Rm*CE and polar contacts were evaluated (Figure S4). Interestingly, the residues responsible for binding of glucose (reducing end of cellobiose) are the same as for galactose, so no clear determinant for monosaccharide activity could be discovered. While all CE enzymes are probably able to recognize monosaccharides in their subsite, it remains unclear why only some representatives display significant activity. Moreover, the monosaccharide specificity among these representatives differs, as evidenced by the activity on galactose by *Rm*CE which was not present in the case of *Cs*CE. 

Interestingly, the *k*_cat_ of the galactose to talose reaction turned out to be relatively high, even though it is only considered a minor activity. The combination of a medium-high activity, a stable enzyme and a cheap substrate led us to believe that a viable production process was possible. Moreover, the product talose, its derivatives and glycoconjugates are valuable molecules with several important industrial applications, like anti-tumor [15] and antimicrobial [33,34] activities as well as recrystallization-inhibition (RI) activity [35]. The only problem in the entire process was the by-product tagatose that was being formed through the isomerization side reaction of the enzyme, similar to the formation of fructose in the production of mannose by *Cs*CE [4]. Therefore, reaction and process optimization were performed to chart the purity and yield of the product by varying substrate concentration and reaction time. This revealed that product purity remained >99% at the beginning of the reaction, but decreased steadily afterwards. In contrast, the product yield kept increasing during the course of the reaction, reaching a maximum yield of about 20%. Upscaling of the reaction was performed and resulted in 23 g/L of d-talose with a purity of 86% and a yield of 8.5%. Depending on the application, a trade-off should be considered between purity and product yield, decreasing reaction time if the former is favored and increasing the reaction time if the latter is preferred. Alternatively, either preparative high-performance liquid chromatography (HPLC) [36,37] or simulated moving bed chromatography might be used to separate monosaccharides, as established by the production of psicose from sucrose with a three-enzyme cascade reaction [9]. A more elegant solution might also be provided by adding an additional enzyme to the reaction mixture, creating a coupled process. This has already been successfully demonstrated in the production of *N*-acetylneuraminic acid, the starting material for anti-influenza agents such as zanamivir and inavir, using an *N*-acyl-d-glucosamine 2-epimerase [38].

In an attempt to increase the activity on monosaccharide substrates, two mutant libraries were constructed and in total 2700 mutants were screened for increased activity. Unfortunately, no improved mutants could be detected, indicating that amino acid positions Tyr-124, Tyr-307, Trp-321 and Trp-385 are not ideal hotspots for gaining *k*_cat_ improvements. Targeting other positions, possibly further away from the active site, could prove to be the key to unlocking higher activities on mannose, galactose and other monosaccharide substrates.

Literature and our own results demonstrated that the *k*_cat_ of *Rm*CE is higher than that of *Cs*CE. As *Cs*CE already holds an application in the production of glucose from mannose, it was decided to try and transfer this property from *Rm*CE to *Cs*CE [4]. Variant S99M/Q371F was able to accomplish this by doubling the *k*_cat_ while more or less retaining the same *K*_m_ value. This shift in activity can probably be attributed to the different orientation of Trp-385 (Trp-372 in *Cs*CE) effectuated by the presence of Phe-384 (Gln-371 in *Cs*CE) and Met-109 (Ser-99 in *Cs*CE).

In conclusion, this paper presents two enzymes that can be applied for industrial production processes: the wild-type *Rm*CE which can efficiently produce d-talose from the cheap substrate d-galactose on the one hand; and a mutant *Cs*CE with a higher *k*_cat_ for the production of d-mannose on the other hand. 

## 4. Materials and Methods 

### 4.1. Gene Cloning of Cellobiose 2-Epimerase 

Cellobiose 2-epimerase from *Rhodothermus marinus* (Uniprot: F8WRK9) was ordered at Genscript and supplied in a pUC57 vector containing a C-terminal His-tag. Next, it was cloned in a pET21a(+) vector acquired from Novagen by making use of the Golden Gate protocol by Engler et al. [39]. The backbone was amplified with forward (5′-GGT CTT CGG TCT CCG TGG CTC GCA TCA TCA TCA T-3′) and reverse primers (5′-GGT CTT CGG TCT CCA TCC CGT ATA TCT CCT TCT TAA AGT-3′) and the gene was amplified with forward (5′-GGT CTT CGG TCT CCG GAT GAG CAC CGA AAC GAT TCC G-3′) and reverse primers (5′-GGT CTT CGG TCT CCC CAC GGG AGC GAA CGT GTT-3′). Then, the resulting plasmid was transformed in *E. coli* BL21(DE3) and plated on lysogeny broth (LB) agar containing 100 μg/mL of ampicillin. An ampicillin-resistant colony was selected and plasmid DNA from the transformant was isolated with a plasmid purification kit (Analytik Jena, Jena, Germany). Afterwards, the plasmid was checked by sequencing (LGC Genomic sequencing service in Berlin, Germany).

### 4.2. Enzyme Production and Purification

The recombinant *E. coli* cells for the expression of *Rm*CE were cultivated in 500 mL of terrific broth (TB) medium in a 2 L flask containing 100 μg/mL of ampicillin. When the optical density of the cells reached 0.6, isopropyl-β-d-thiogalactopyranoside (IPTG) was added to a final concentration of 0.1 mM and the culture was incubated shaking at 200 rpm at 30 °C for 16 h. The produced biomass was harvested by centrifugation at 4 °C for 20 min at 4500 rpm and the resulting pellet was frozen at −20 °C for at least 16 h. For enzyme extraction and purification, cell pellets were thawed and dissolved in 10 mL lysis buffer (300 mM NaCl, 10 mM imidazole, 0.1 mM PMSF and 50 mM sodium phosphate buffer; pH 7.4) supplemented with lysozyme at a final concentration of 1 mg/mL. The resuspended cells were incubated on ice for 30 min and sonicated two times for 4 min (Branson Sonifier 250, level 3, 50% duty cycle). Next, cell lysate was centrifuged two times for 15 min at 14,000 rpm.

Proteins from the soluble supernatant fraction were purified by Ni-NTA chromatography as described by the supplier (Thermo Scientific, Waltham, MA, USA), after which the buffer was exchanged to 50 mM MOPS buffer (pH 6.3) in an Amicon® Ultra centricon with a 30 kDa cut-off (Merck Millipore, Darmstadt, Germany). The protein content was determined by measuring the absorbance at 280 nm with the NanoDrop2000 Spectrophotometer (Thermo Scientific). The extinction coefficient and molecular weight of His-tagged *Rm*CE were calculated using the ProtParam tool on the ExPASy server (http://web.expasy.org/protparam/). Molecular weight and purity of the protein were verified by sodium dodecyl sulfate polyacrylamide gel electrophoresis (SDS-PAGE; 12% gel).

### 4.3. Substrate Specificity Analysis

The specificity of *Rm*CE towards different monosaccharides was determined in MOPS buffer (pH 6.3) and with 200 mM of substrate (d-glucose, d-mannose, d-galactose, d-xylose, d-lyxose, l-arabinose) at an enzyme concentration of 0.3 mg/mL. All reactions were monitored at 70 °C and 5 time points were taken during 24 h. Enzyme inactivation was achieved by transferring the sample (5 μL) in 100 mM of NaOH (90 μL). Conversion of substrate to product was evaluated by high-performance anion exchange chromatography–pulsed amperometric detection (HPAEC–PAD) using the Dionex ICS-3000 system (Thermo Fischer Scientific) (CarboPac PA20 column-3 × 150 mm) as described by Verhaeghe et al. but with a 15-min isocratic method of 20 mM NaOH [40]. The monosaccharides were quantified by determining conversion based on the peak areas. Product concentration was then plotted in function of time and a linear correlation was fitted representing the activity of the enzyme (in μmol/min). Finally, specific activities were calculated by dividing the increase in product (units) by the enzyme concentration (mg). One unit (U) of *Rm*CE activity was defined as the amount of enzyme required to produce 1 μmol of product from substrate per minute at 70 °C and pH 6.3.

### 4.4. Kinetic Parameters of RmCE

The kinetic parameters for mannose were determined with a discontinuous coupled assay using glucose oxidase and peroxidase (GOD-POD) as described by Aerts et al. [41]. Since the assay can, to a small extent, also detect mannose, different standard series were created mimicking the formation of glucose in different starting mannose concentrations (Appendix A). Reactions were performed in 100 mM MOPS (pH 6.3) and monitored at 70 °C during 90 min. Five time points were taken for each mannose concentration (50, 100, 200, 300, 400, 750, 1000 mM) and activities were determined in the same way as in Section 4.3. 

Since the GOD-POD method was not applicable for detection of talose, the Dionex ICS-3000 system (Thermo Fischer Scientific) (CarboPac PA20 column-3 × 150 mm) was used for detection of galactose and its products tagatose and talose. Samples were diluted to a concentration of approximately 100 μM and analysed at a flow rate of 0.5 mL/min. Separation of galactose, tagatose and talose was accomplished by isocratic elution with 8 mM of NaOH for 12 min, followed by a linear gradient for 13 min from 8 mM to 45 mM of NaOH. Afterwards, initial conditions were restored in 1 min and maintained for 10 min. Enzyme reactions were performed in 100 mM MOPS (pH 6.3) and monitored at 70 °C during 90 min. Six time points were taken for each galactose concentration (200, 400, 800, 1200, 1600 mM) and activities were determined in the same way as in Section 4.3. The kinetic parameters, including the Michaelis–Menten constant (*K*_m_) and turnover number (*k*_cat_), were determined using Michaelis–Menten plots created in SigmaPlot (Systat Software, San Jose, CA, USA).

### 4.5. Talose Production

First of all, two parameters (substrate concentration and incubation time) were optimized to maximize yield and purity. Unless otherwise stated, reactions were performed at 70 °C in 100 mM MOPS (pH 6.3) with an *Rm*CE concentration of 0.3 mg/mL. To evaluate the effect of the substrate on the purity of the galactose/tagatose/talose mixture, five different galactose concentrations (200, 400, 800, 1200, 1600 mM) were tested by collecting seven time points during 18 h of reaction. Subsequently, product purity ratios at each time point were determined more precisely by performing the reaction at 1.6 M of galactose in triplicate and were calculated with the formula (Tal)/((Tal)+(Tag)). In addition, yield was determined by the formula (Tal)/((Gal)+(Tal)+(Tag)) and displayed in percentage. Conversion of galactose to talose and tagatose was evaluated by HPAEC–PAD using the same method and settings as described in Section 4.4.

Next, a large-scale reaction was perfomed to evaluate whether it was possible to get larger amounts of product with acceptable purity. 15 mL of reaction mixture containing 4.3 g (24 mmol) of galactose, 0.3 mg/mL of His-tag purified *Rm*CE and 100 mM of MOPS (pH 6.3) was incubated at 70 °C and the reaction was stopped after 4.5 h by incubation for 10 min at 95 °C. Protein debris was removed by centrifugation for 20 min at 18,000 rpm and filtration through a polyethersulfone membrane with a cut-off of 0.2 μm. 

To remove galactose from the mixture, *Kluyveromyces marxianus* was grown in 50 mL of medium (yeast extract, 10 g/L; peptone, 20 g/L; galactose, 20g/L) at 200 rpm and 30 °C until at least an OD600 of 6 was reached. This particular strain has the advantage of consuming galactose whereas a typical strain like *Saccharomyces cerevisiae* would not [20,21]. Moreover, the glycerol production is very low, even after long incubation periods. The yeast was pregrown with galactose as primary carbon source instead of glucose allowing the metabolism to already adapt towards galactose consumption. This speeds up the subsequent process of removing galactose in the reaction mixture. Then, the cell suspension was centrifuged for 20 min at 4500 rpm and the yeast cells were washed with MilliQ water (ELGA LabWater, High Wycombe, UK) once and afterwards resuspended in 5 mL of 100 mM MOPS (pH 7). Half of this suspension was added to the filtrated reaction mixture (diluted until a theoretical concentration of 20 g/L monosaccharides) and incubated for 96 h at 30 °C and 200 rpm and centrifuged for 20 min at 4500 rpm. Finally, the supernatant was evaporated for 3 h and saved at −20 °C. The purity of the final product was evaluated by analysis with Dionex as described in Section 4.4. The amount of talose was estimated by a standard curve with talose obtained from Carbosynth (Compton, UK).

### 4.6. Developing and Screening Two Mutant Libraries to Increase Activity on Monosaccharide Substrates

Exploration of the active site of *Rm*CE, based on the crystal structure (PDB code: 3WKG), revealed four interesting positions for mutagenesis: Tyr-124, Tyr-307, Trp-321 and Trp-385. Since complete saturation of these positions is not possible, as this would deliver too big a library, the Rosetta suite was considered as alternative. The full procedure can be retrieved in Appendix B.

#### 4.6.1. Generation of Mutant Libraries

Two mutant libraries (short_sim and long_sim) were created by circular polymerase extension cloning (CPEC) [42] with different amino acids on the four targeted positions. Four prefragmens were generated starting from the pET21a-*Rm*CE WT construct using the primers from Appendix C.1. This step was necessary to ensure that the distribution of the introduced amino acids was equal and not favored towards the wild-type amino acid. The actual CPEC fragments were constructed by performing an additional polymerase chain reaction (PCR) with the prefragments and the degenerated primers from Appendix C.2 and Appendix C.3. These primers were designed to have an overhang that displays an overlap with a melting temperature of 63–64 °C which is important to increase the efficiency of the CPEC reaction. Afterwards, a *Dpn*I digest for 3 h at 37 °C was performed on all PCR-fragments. After pooling of the fragments in the final PCR reaction, the resulting plasmid libraries of both short_sim and long_sim were transformed in electrocompetent *E. coli* BL21(DE3) cells. Next, the transformation mixture was transferred to LB medium containing 100 μg/mL ampicillin and plasmid DNA was isolated with a plasmid purification kit (Analytik Jena). Afterwards, the distribution of the incorporated nucleotides on each of the four codon positions was checked by sequencing (LGC Genomic sequencing service in Berlin, Germany).

#### 4.6.2. Screening of Mutant Libraries

Library plasmids were again transformed in electrocompetent *E. coli* BL21(DE3) cells and plated on LB medium containing 100 μg/mL ampicillin. Colonies from this plate were transferred to microtiterplates containing 175 μL LB medium complemented with 100 μg/mL ampicillin. After 16 h incubation at 37 °C and 250 rpm, 10 μL from each well was transferred to a new microtiterplate containing 170 μL TB medium complemented with 100 μg/mL ampicillin. After growth for two hours at 37 °C and 250 rpm the mutants were induced with 0.1 mM IPTG. Then, after incubation for 16 h at 30 °C and 250 rpm, cells were harvested by centrifugation for 20 min at 4500 rpm. Pellets were frozen at −20 °C for at least 1 h and resuspended in 175 μL lysis buffer (50 mM MOPS (pH 6.3), 1 mg/mL lysozym, 0.1 mM phenylmethylsulfonyl fluoride (PMSF), 1 mM ethylenediaminetetraacetic acid (EDTA), 4 mM MgCl_2_ and 50 mM NaCl). Cell lysis went on for 30 min at 37 °C. Subsequently, 25 μL of a mannose stock solution was added to a final concentration of 100 mM. After 1h a sample from each well was taken and analyzed by the GOD-POD assay. Finally, the absorbance in the wells containing the mutants was compared to the mean wild-type absorbance. A mutant enzyme was considered a hit if the following condition was fulfilled: absorbance (mutant) > mean absorbance (WT) + mean absorbance (WT) × CV, where CV is the coefficient of variation. The CV was calculated by taking the standard deviation of the absorbance of 48 microtiter plate wells with wild-type and dividing by the mean signal of these wells. To obtain 95% coverage of each library, 21 plates were screened for short_sim and six plates were screened for long_sim.

### 4.7. Cloning of CsCE and S99M/Q371F Variant, Enzyme Expression, Purification and Activity Tests

Cellobiose 2-epimerase from *Caldicellulosiruptor saccharolyticus* (Uniprot: A4XGA6) was ordered and subcloned in the pET21a(+) vector at GeneArt. The S99M/Q371F variant was created starting from this wild-type plasmid using forward (5′-GCCTGTTTTGGATGGTTAGCCATAAAGG-3′) and reverse primers (5′-GGGCATTTCCAAAATTCAACAATCGGCAT-3′) with the method devoped by Sanchis et al. [43]. Expression and purification of the *Cs*CE wild-type and variant enzyme was performed as described for *Rm*CE in Section 4.2. Activity tests were performed with 200 mM of mannose and 1 mg/mL enzyme in 100 mM of MOPS pH 7.5. Reactions were incubated at 70 °C and six time points were taken during 20 min. Enzyme inactivation was achieved by transferring the sample (5 μL) in 100 mM of NaOH (95 μL). Samples were properly diluted and analyzed by the GOD-POD assay (Section 4.4).

## Figures and Tables

**Table 1:** Substrate specificity of *Rhodothermus marinus* (*Rm*CE). Specific activities were determined on 200 mM substrate at pH 6.3 and 70 °C. All tests were performed in triplicate and the standard deviation is reported for every substrate. No activity could be detected on l-arabinose.

Substrate	Product	*Rm*CE Specific Activity (mU/mg)	*Cs*CE Specific Activity (mU/mg) ^1^
Cellobiose	4-*O*-β-d-glucopyranosyl-d-mannose	87,500 ^2^	3600 ± 2.1
d-mannose	d-glucose	1130 ± 25	150 ± 0.5
d-glucose	d-mannose	440 ± 24	35 ± 0.5
d-galactose	d-talose	1020 ± 83	/
d-xylose	d-lyxose	38 ± 5	40 ± 0.7
d-lyxose	d-xylose	18 ± 0.2	60 ± 0.3
l-arabinose	l-ribose	/	/

^1^ Data represented as described in Park et al [4]. ^2^ Data obtained from Ojima et al [5].

**Table 2:** Kinetic parameters of cellobiose 2-epimerase from *R. marinus* for d-mannose and d-galactose. Tests were performed at pH 6.3 and 70 °C. Data represent the mean of three experiments with standard error.

Substrate	*K_m_* (M)	*k_cat_*(s^−1^)	*k_ca_*_t_/*K_m_*(s^−1^/M^−1^)
d-mannose	0.45 ± 0.18	5.4 ± 1	12.0 ± 4.4
d-galactose	1.04 ± 0.13	5.0 ± 0.3	4.8 ± 0.7

**Table 3:** Time-course representation of galactose to talose conversion depicting product purity, yield and concentration. Tests were performed at a substrate concentration of 1.6 M and with an enzyme concentration of 0.3 mg/mL. Data represent the means of three experiments with standard deviation.

Time (h)	Purity (%)	Yield (%)	d-talose (mM)
0.5	>99	0.50 ± 0.30	8.07 ± 4.85
1	>99	1.90 ± 0.16	30.36 ± 2.49
1.5	97.01 ± 2.59	2.68 ± 0.23	42.81 ± 3.76
3	93.65 ± 1.92	5.43 ± 0.08	86.90 ± 1.36
4.5	92.47 ± 1.26	8.97 ± 0.66	143.50 ± 10.50
7	91.22 ± 0.86	12.01 ± 0.82	192.14 ± 13.18
18	89.78 ± 1.53	19.28 ± 1.28	308.40 ± 20.44

**Table 4:** Kinetic parameters of cellobiose 2-epimerase from *C. saccharolyticus* for d-mannose, both for wild-type and double mutant S99M/Q371F. Tests were performed at pH 7.5 and 70 °C. Data represent the mean of three experiments with standard error.

*Cs*CE	*K*_m_ (mM)	*k*_cat_ (s^−1^)
WT	412 ± 91	3.8 ± 0.2
S99M/Q371F	430 ± 94	7.5 ± 0.5

## Supplementary Materials

The following are available online. Table S1: Substrate concentration optimization of the *Rm*CE reaction with galactose performed at pH 6.3 and 70 °C; Figure S1: Time-course representation of *Kluyveromyces marxianus *treatment of the upscaled *Rm*CE reaction for talose production; Figure S2: Dionex profile of the final dried mixture containing glycerol (Gly), galactose (Gal), tagatose (Tag) and talose (Tal); Figure S3: Overview of the introduced amino acids on each position in both libraries. Figure S4: Active site representation of *Rm*CE (PDB code: 3WKG) containing docked galactose (element = blue) as substrate.

## Appendix A

**Table A1:** Standard series employed during the GOD-POD assays. The ratios represent the amount of mannose and glucose present in the different standards (mm mannose:mm glucose).

50 mM Std	100 mM Std	200 mM Std	300 mM Std	400 mM Std	750 mM Std	1 M Std
50:0	100:0	200:0	300:0	400:0	750:0	1000:0
49:1	98:2	198:2	295:5	395:5	740:10	990:10
48:2	96:4	195:5	290:10	390:10	730:20	980:20
47:3	94:6	192:8	280:20	380:20	710:40	970:30
46:4	92:8	190:10	275:25	370:30	700:50	950:50
45:5	90:10	180:20	270:30	360:40	690:60	930:70

## Appendix B. Employing Rosetta EnzDes to Determine Useful Amino Acid Substitutions on Target Positions

The *Rm*CE structure (3WKG) was subjected to a molecular dynamics simulation to generate starting structures for the Rosetta suite (all steps were performed with the Yasara Structure software). Two separate simulations of different simulation times (0.1 ns and 5 ns) were executed and five snapshots were taken at regular time intervals. First of all, the structure was prepared for the molecular dynamics simulation by employing the ‘clean structure’ function. Then, a simulation cell 5 Å around all atoms was defined and this box was filled with water atoms and, accordingly, neutralized by ions if necessary. Subsequently, an energy minimization was performed until the mean velocity dropped below 3000 m/s after which ‘simulated annealing’ mode was used until temperature dropped below 30 K. Finally, the actual simulation was performed with the ‘rescale velocity’ function and following characteristics: temperature was constant at 298 K, pressure control was enabled and solvent molecules remained at a constant water density/pressure.

Ligand input files (consisting of a pdb and a .params for each conformer) and constraint files were constructed. The catalytic constraints are specific parameters (like distance and angle) between atoms of the catalytic residues and ligand atoms and represent the borders within which decent catalysis can still occur. Four residues (Tyr-124, Tyr-307, Trp-321 and Trp-385) were defined as designable, while all other residues within 7 Å of the ligand were defined as repackable.

The Rosetta EnzDes suite [26,44] was run 500 times per starting structure, generating 2500 possibly improved structures for both the short and the long simulation. All these structures were ranked by Rosetta based on the ΔΔG value representing the difference in binding energy between the ligand and the designed protein against the wild type. Furthermore, structures displaying a constraint value, representing the fit of the ligand to the catalytic residues, higher than 30 were omitted from further analysis. Finally, the introduced amino acids on each designable position were evaluated and amino acids occurring in more than 5% of the remaining sequencing were considered in the generation of a mutant library.

## Appendix C

### Appendix C.1. Primers for the Amplification of Prefragments

**Table:**

Primer Name	Primer
*Rm*CE_bib_A125_Fw	GCCCAGAGTTTTGCTATC
*Rm*CE_bib_Y306_Rv	ATACAGAGAGCCATCCG
*Rm*CE_bib_W322_Fw	TGGCCGCAAGCCGAA
*Rm*CE_bib_F384_Rv	GAAATCGACTTTGTCATCCG
*Rm*CE_bib_K386_Fw	AAAGGTCCGTATCATAATGGC
*Rm*CE_bib_V123_Rv	AACATGTTTACGGGTGTC

### Appendix C.2. Primers for the Long Library Fragments

**Table:**

Primer Name	Primer
*Rm*CE_bib_Y124Y_Fw	CCGCTGGACACCCGTAAACATGTTTATGCCCAGAGTTTTGCTATC
*Rm*CE_bib_Y124P_Fw	CCGCTGGACACCCGTAAACATGTTCCGGCCCAGAGTTTTGCTATC
*Rm*CE_bib_Y124V_Fw	CCGCTGGACACCCGTAAACATGTTGTGGCCCAGAGTTTTGCTATC
*Rm*CE_bib_Y307YH_Rv	GTCGGTATCCAGATGGCCCTGTTCACCAATTTCATRATACAGAGAGCCATCCG
*Rm*CE_bib_Y307K_Rv	GTCGGTATCCAGATGGCCCTGTTCACCAATTTCTTTATACAGAGAGCCATCCG
*Rm*CE_bib_W321WR_Fw	GTGAACAGGGCCATCTGGATACCGACCGTCACYGGTGGCCGCAAGCCGAA
*Rm*CE_bib_W321A_Fw	GTGAACAGGGCCATCTGGATACCGACCGTCACGCGTGGCCGCAAGCCGAA
*Rm*CE_bib_W321M_Fw	GTGAACAGGGCCATCTGGATACCGACCGTCACATGTGGCCGCAAGCCGAA
*Rm*CE_bib_W385WR_Rv	CGCGGCCATTATGATACGGACCTTTCCRGAAATCGACTTTGTCATCCG
*Rm*CE_bib_W385T_Rv	CGCGGCCATTATGATACGGACCTTTGGTGAAATCGACTTTGTCATCCG
*Rm*CE_bib_W385YD_Rv	CGCGGCCATTATGATACGGACCTTTATMGAAATCGACTTTGTCATCCG

### Appendix C.3. Primers for the Short Library Fragments

**Table:**

Primer Name	Primer
*Rm*CE_bib_Y124YH_Fw	CCGCTGGACACCCGTAAACATGTTYATGCCCAGAGTTTTGCTATC
*Rm*CE_bib_Y124A_Fw	CCGCTGGACACCCGTAAACATGTTGCGGCCCAGAGTTTTGCTATC
*Rm*CE_bib_Y124L_Fw	CCGCTGGACACCCGTAAACATGTTCTGGCCCAGAGTTTTGCTATC
*Rm*CE_bib_Y307YH_Rv	GTCGGTATCCAGATGGCCCTGTTCACCAATTTCATRATACAGAGAGCCATCCG
*Rm*CE_bib_Y307K_Rv	GTCGGTATCCAGATGGCCCTGTTCACCAATTTCTTTATACAGAGAGCCATCCG
*Rm*CE_bib_Y307R_Rv	GTCGGTATCCAGATGGCCCTGTTCACCAATTTCGCGATACAGAGAGCCATCCG
*Rm*CE_bib_Y307A_Rv	GTCGGTATCCAGATGGCCCTGTTCACCAATTTCCGCATACAGAGAGCCATCCG
*Rm*CE_bib_W321A_Fw	GTGAACAGGGCCATCTGGATACCGACCGTCACGCGTGGCCGCAAGCCGAA
*Rm*CE_bib_W321M_Fw	GTGAACAGGGCCATCTGGATACCGACCGTCACATGTGGCCGCAAGCCGAA
*Rm*CE_bib_W321YH_Fw	GTGAACAGGGCCATCTGGATACCGACCGTCACYATTGGCCGCAAGCCGAA
*Rm*CE_bib_W321W_Fw	GTGAACAGGGCCATCTGGATACCGACCGTCACTGGTGGCCGCAAGCCGAA
*Rm*CE_bib_W385KE_Rv	CGCGGCCATTATGATACGGACCTTTTTYGAAATCGACTTTGTCATCCG
*Rm*CE_bib_W385WR_Rv	CGCGGCCATTATGATACGGACCTTTCCRGAAATCGACTTTGTCATCCG
*Rm*CE_bib_W385T_Rv	CGCGGCCATTATGATACGGACCTTTGGTGAAATCGACTTTGTCATCCG
*Rm*CE_bib_W385YD_Rv	CGCGGCCATTATGATACGGACCTTTATMGAAATCGACTTTGTCATCCG
