# Supplementary material for: Converting Galactose into the Rare Sugar Talose with Cellobiose 2-Epimerase as Biocatalyst
Source: Molecules. 2018 Oct 1;23(10):2519. doi: 10.3390/molecules23102519 (PMC6222537; doi:10.3390/molecules23102519)
Supplement: Supplementary file 1 [file molecules-23-02519-s001.zip › Supplementary data/Figure S1.pdf]

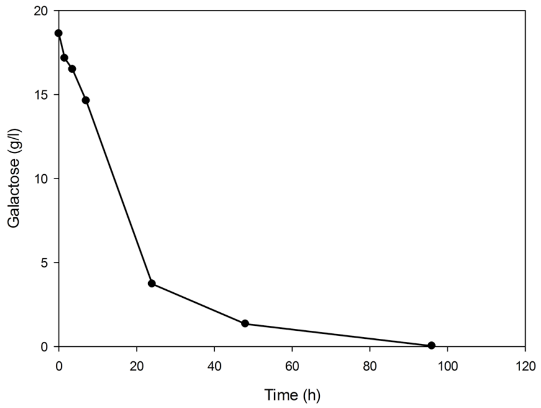

Figure S1: Time-course representation of *Kluyveromyces* treatment of the upscaled *Rm*CE reaction for talose production.
