# Supplementary material for: Converting Galactose into the Rare Sugar Talose with Cellobiose 2-Epimerase as Biocatalyst
Source: Molecules. 2018 Oct 1;23(10):2519. doi: 10.3390/molecules23102519 (PMC6222537; doi:10.3390/molecules23102519)
Supplement: Supplementary file 1 [file molecules-23-02519-s001.zip › Supplementary data/Figure S2.pdf]

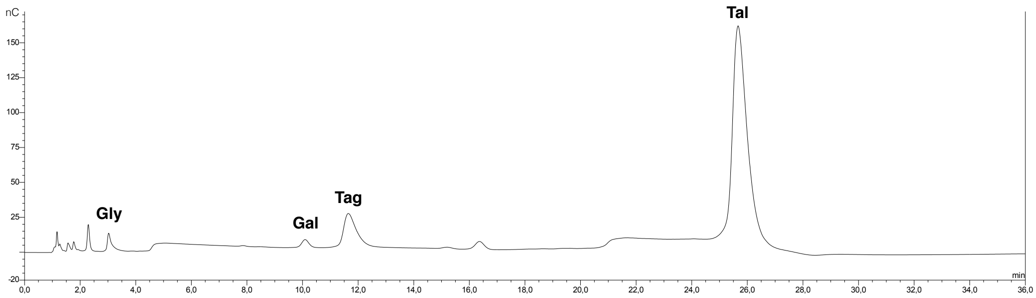

Figure S2: Dionex profile of the final dried mixture containing glycerol (Gly), galactose (Gal), tagatose (Tag) and talose (Tal).
