# Supplementary material for: Converting Galactose into the Rare Sugar Talose with Cellobiose 2-Epimerase as Biocatalyst
Source: Molecules. 2018 Oct 1;23(10):2519. doi: 10.3390/molecules23102519 (PMC6222537; doi:10.3390/molecules23102519)
Supplement: Supplementary file 1 [file molecules-23-02519-s001.zip › Supplementary data/Figure S3.pdf]

Short simulation (0,1 ns)				Long simulation (5 ns)			
Y124	Y307	W321	W385	Y124	Y307	W321	W385
Y	Y	W	W	Y	Y	W	W
A	R	A	T	P	H	A	T
H	H	M	Y	V	K	M	Y
L	A	H	D			R	D
	K	Y	K				R
			E				
			R				

Figure S3: Overview of the introduced amino acids on each position in both libraries. The figure represents two libraries generated by Rosetta based on starting structures originating from a molecular dynamics simulation of 0.1 and 5 ns, respectively. Residues are only displayed when they occurred in more than 5% of the sequences on a given position.
