# Supplementary material for: Converting Galactose into the Rare Sugar Talose with Cellobiose 2-Epimerase as Biocatalyst
Source: Molecules. 2018 Oct 1;23(10):2519. doi: 10.3390/molecules23102519 (PMC6222537; doi:10.3390/molecules23102519)
Supplement: Supplementary file 1 [file molecules-23-02519-s001.zip › Supplementary data/Figure S4.pdf]

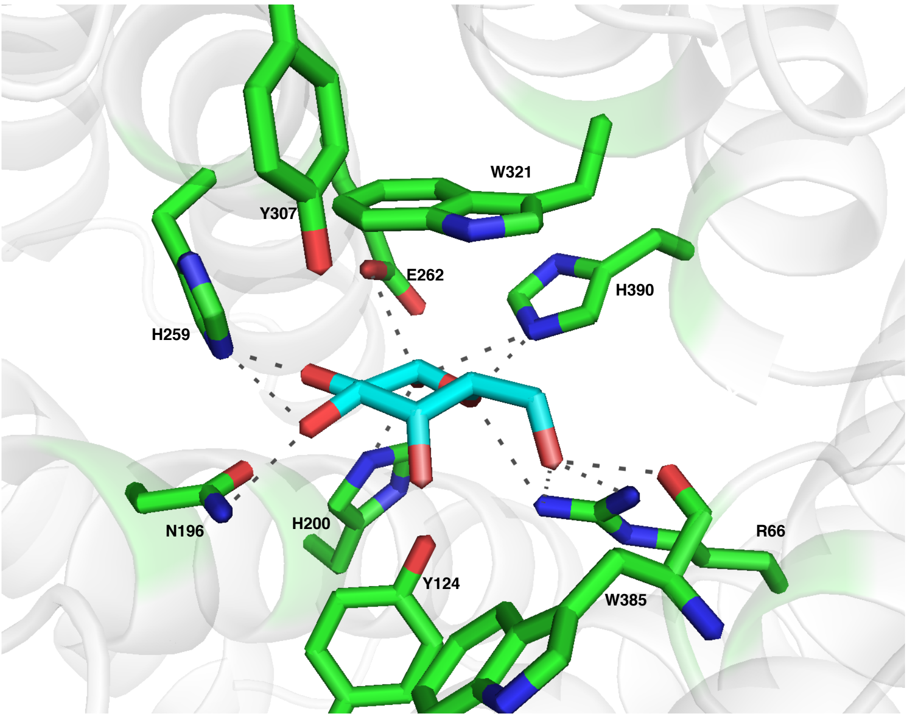

Figure S4: Active site representation of RmCE (PDB: 3WKG) containing docked galactose (element=blue) as substrate. Side-chain residues making polar contacts with the substrate (R66, N196, H200, H259, E262, H390) and the mutagenesis targets (Y124, Y307, W321, W385) are displayed and colored by element green. The same residues are responsible for substrate binding in comparison to the glucose moiety of cellobiose.
