# Supplementary material for: Converting Galactose into the Rare Sugar Talose with Cellobiose 2-Epimerase as Biocatalyst
Source: Molecules. 2018 Oct 1;23(10):2519. doi: 10.3390/molecules23102519 (PMC6222537; doi:10.3390/molecules23102519)
Supplement: Supplementary file 1 [file molecules-23-02519-s001.zip › Supplementary data/Table S1.pdf]

Table S1: Substrate concentration optimization of the *Rm*CE reaction with galactose performed at pH 6.3 and 70°C.

Time (h)	200mM		400mM		800mM		1,2M		1,6M	
	Pur(%)	Tal(mM)	Pur(%)	Tal(mM)	Pur(%)	Tal(mM)	Pur(%)	Tal(mM)	Pur(%)	Tal(mM)
0.5	>99	2.62±0.41	>99	5.40±0.21	>99	9.55±0.42	>99	9.76±1.22	>99	8.07±4.85
1	>99	5.20±0.16	>99	9.41±0.58	>99	20.32±1.29	>99	17.03±1.10	>99	30.36±2.49
1.5	96.30±2.05	9.69±0.15	95.27±3.68	19.09±0.25	95.56±2.27	35,16±0.88	94.57±3,75	30.62±2.20	97.01±2.59	42.81±3.76
3	94.03±1.20	21.20±0.77	93.33±1.12	36.05±1.01	91.86±2.21	61,17±4.30	92.34±2.30	63.77±6.46	93.65±1.92	86.90 1.36
4.5	92.83±1.29	25.58 ±			91.45±3.58	76.13 ±	90.94±2.24	98.29±10.42		
		0.33	91.70±1.78	44.40±1.32		6.16			92.47±1.26	143.50±10.50
7	88.53	31.65	88.68	59.07	88.22	103.00	89.17	116.77	91.22±0.86	192.14±13.18
18	73.95	45.72	77.80	86.41	78.52	169.50	86.83	205.99	89.78±1.53	308.40±20.44
